# The RELIEF feasibility trial: topical lidocaine patches in older adults with rib fractures

**DOI:** 10.1136/emermed-2024-213905

**Published:** 2024-05-16

**Authors:** Madeleine Clout, Nicholas Turner, Clare Clement, Philip Braude, Jonathan Benger, James Gagg, Emma Gendall, Simon Holloway, Jenny Ingram, Rebecca Kandiyali, Amanda Lewis, Nick A Maskell, David Shipway, Jason E Smith, Jodi Taylor, Alia Darweish Medniuk, Edward Carlton

**Affiliations:** 1 Population Health Sciences, University of Bristol, Bristol, UK; 2 University of the West of England, Bristol, UK; 3 CLARITY (Collaborative Ageing Research), North Bristol NHS Trust, Westbury on Trym, UK; 4 Faculty of Health and Life Sciences, University of the West of England, Bristol, UK; 5 Department of Emergency Medicine, Somerset NHS Foundation Trust, Taunton, UK; 6 Research and Innovation, Southmead Hospital, Bristol, UK; 7 Pharmacy Clinical Trials and Research, Southmead Hospital, Bristol, UK; 8 Bristol Medical School, University of Bristol, Bristol, UK; 9 Warwick Clinical Trials Unit, Warwick Medical School, Coventry, UK; 10 Academic Respiratory Unit, University of Bristol, Bristol, UK; 11 Department of Medicine for Older People, Southmead Hospital, North Bristol NHS Trust, Bristol, UK; 12 Emergency Department, University Hospitals Plymouth NHS Trust, Plymouth, UK; 13 Bristol Trials Centre, Population Health Sciences, University of Bristol, Bristol, UK; 14 Department of Anaesthesia and Pain Medicine, Southmead Hospital, Bristol, UK; 15 Emergency Department, Southmead Hospital, Bristol, UK; 16 Department of Emergency Medicine, Translational Health Sciences, University of Bristol, Bristol, UK

**Keywords:** local, feasibility studies, frail elderly, chest, geriatrics

## Abstract

**Background:**

Lidocaine patches, applied over rib fractures, may reduce pulmonary complications in older patients. Known barriers to recruiting older patients in emergency settings necessitate a feasibility trial. We aimed to establish whether a definitive randomised controlled trial (RCT) evaluating lidocaine patches in older patients with rib fracture(s) was feasible.

**Methods:**

This was a multicentre, parallel-group, open-label, feasibility RCT in seven hospitals in England and Scotland. Patients aged ≥65 years, presenting to ED with traumatic rib fracture(s) requiring hospital admission were randomised to receive up to 3×700 mg lidocaine patches (Ralvo), first applied in ED and then once daily for 72 hours in addition to standard care, or standard care alone. Feasibility outcomes were recruitment, retention and adherence. Clinical end points (pulmonary complications, pain and frailty-specific outcomes) and patient questionnaires were collected to determine feasibility of data collection and inform health economic scoping. Interviews and focus groups with trial participants and clinicians/research staff explored the understanding and acceptability of trial processes.

**Results:**

Between October 23, 2021 and October 7, 2022, 206 patients were eligible, of whom 100 (median age 83 years; IQR 74–88) were randomised; 48 to lidocaine patches and 52 to standard care. Pulmonary complications at 30 days were determined in 86% of participants and 83% of expected 30-day questionnaires were returned. Pulmonary complications occurred in 48% of the lidocaine group and 59% in standard care. Pain and some frailty-specific outcomes were not feasible to collect. Staff reported challenges in patient compliance, unfamiliarity with research measures and overwhelming the patients with research procedures.

**Conclusion:**

Recruitment of older patients with rib fracture(s) in an emergency setting for the evaluation of lidocaine patches is feasible. Refinement of data collection, with a focus on the collection of pain, frailty-specific outcomes and intervention delivery are needed before progression to a definitive trial.

**Trial registration number:**

ISRCTN14813929.

**WHAT IS ALREADY KNOWN ON THIS TOPIC**

- Studies have evaluated the use of lidocaine patches in patients with rib fractures showing reductions in opioid use, improvements in pain scores and reductions in length of hospital stay.
- Importantly, none has focused on older patients, who stand to gain the most benefit from improved analgesic regimens to reduce adverse pulmonary complications.

**WHAT THIS STUDY ADDS**

- In this feasibility trial, prespecified progression criteria around recruitment, follow-up and adherence were met, demonstrating it is feasible to conduct randomised controlled trials in older patients, who are in pain, in an emergency setting.
- There were challenges in data collection for pain and frailty-specific measures, together with treatment crossover, that require consideration in definitive trial design.

**HOW THIS STUDY MIGHT AFFECT RESEARCH, PRACTICE OR POLICY**

- Researchers can adapt study processes to be inclusive of older patients in the emergency setting.
- There are challenges in terms of data collection around pain and frailty-specific outcome measures which future research should consider.

## Introduction

Rib fractures represent the most common non-spinal fracture in older people.^1^ Age ≥65 years remains a predictor of morbidity and mortality in patients with rib fractures.^2^ Pain can compromise normal respiratory function, with over 15% of older patients experiencing complications including pneumonia and death.^3^

The mainstay for treatment of rib fracture pain remains strong opioid analgesia. However, as a result of poor physiological reserve, older patients are more vulnerable than younger people to the side effects of strong opioid medication such as nausea, constipation, sedation, delirium and respiratory depression.^4^ Invasive approaches, such as thoracic epidural anaesthesia, have been used to reduce the likelihood of these side effects, but require specialist anaesthetic support, monitoring in a high-dependency environment and are only used in around 20% of admitted patients.^5 6^

Lidocaine patches applied over rib fractures have been suggested as a non-invasive method of local anaesthetic delivery to improve respiratory function, reduce opioid consumption and consequently reduce pulmonary complications.^7^ Studies have evaluated the use of lidocaine patches in patients with rib fractures showing reductions in opioid use,^8^ improvements in pain scores^9 10^ and reductions in length of hospital stay.^11^ However, these studies are limited by retrospective design and low patient numbers with consequent bias and low precision. Importantly, none has focused on older patients, who are more susceptible to the development of pulmonary complications,^2^ or tested lidocaine patches as an intervention in the ED where opioid analgesia is the mainstay of treatment.

Older people have often been excluded from research, relating to multiple long-term health conditions, social and cultural barriers and potentially impaired capacity to provide informed consent.^12^ In addition, recruitment of older patients who are in pain in an emergency setting may pose further challenges around information provision and collection of clinical and patient-reported outcomes.

The aim of this trial was to establish whether a definitive randomised controlled trial (RCT) to evaluate the benefit of lidocaine patches, first applied in the ED, for older people requiring admission to hospital with rib fracture(s) is feasible.

## Methods

Detailed methods, including detailed consent procedures, are described in full elsewhere.^13^

### Design, setting and participants

**Figure 1:**
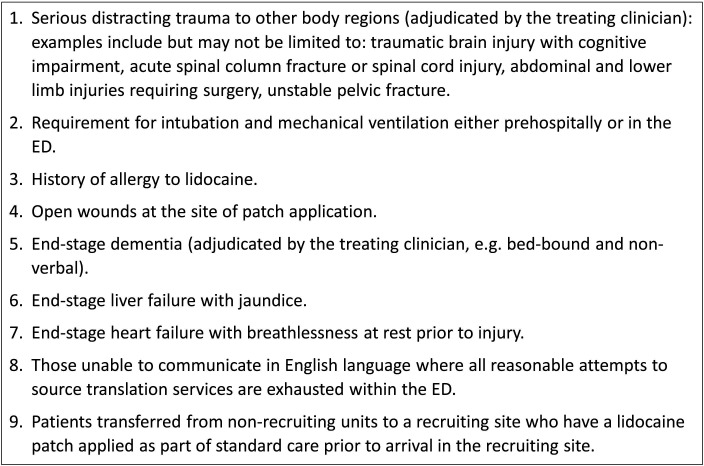
Exclusion criteria.

The Randomised Evaluation of topical Lidocaine patches in Elderly patients admitted to hospital with rib Fractures (RELIEF) study was a multicentre, parallel-group, open-label, individually randomised, feasibility RCT, conducted in seven NHS hospitals: five major trauma centres (Southmead Hospital; Royal Infirmary of Edinburgh; Derriford Hospital, Plymouth; Queen Elizabeth University Hospital, Glasgow; St George’s Hospital, London) and two trauma units (Musgrove Park Hospital, Taunton; Royal Devon and Exeter Hospital). The trial included a health economic scoping analysis and an integrated qualitative study. Patients were eligible for recruitment if they were aged ≥65 years, presented at any time after injury with traumatic rib fracture(s) (including multiple fractures, flail chest and traumatic haemothorax/pneumothorax even if this required intercostal chest drainage), confirmed radiologically (by CXR or CT conducted as part of routine care) and required hospital admission for ongoing care. Exclusion criteria are detailed in figure 1.

### Randomisation and blinding

Participants were randomised in the ED by trained research or clinical staff, using an online randomisation system, with the randomisation sequence generated by Sealed Envelope (London, UK). Participants were allocated to the intervention or standard care in a 1:1 ratio. Randomisation was stratified by trial site and gender and blocked within strata. Allocations were blinded only to those performing central review of data for the assessment of outcomes.

### Intervention

Participants randomised to the intervention received up to 3×700 mg lidocaine patches (Ralvo) at a time applied over the most painful area of rib injury. Patches were first applied in the ED, then once daily for 12 hours in accordance with the manufacturer’s (Grünenthal, Aachen, Germany) instructions. Treatment continued for up to 72 hours or until discharge from hospital. The intervention was additive to standard care (below). If participants subsequently underwent regional anaesthesia, patches were removed and no further patches were applied but data collection continued according to group allocation.

### Standard care

All participants received standard local analgesic treatment for patients with rib fractures; this was not controlled for trial purposes. Data were collected on paracetamol, weak opioid, strong opioid and other non-opioid analgesia prescriptions in ED and for the 72-hour intervention period in both arms of the trial.^14^

### Patient and public involvement

Patient and public involvement was ensured at all stages of trial design, and continued throughout the trial’s lifetime via a patient advisory group and patient representation on the trial steering committees.

### Clinical outcomes and measurement

Outcomes were measured at baseline, 72 hours (during or on completion of intervention) and 30-day postrandomisation. A full schedule of clinical data, questionnaires and end points is included in the published protocol.^13^ Clinical end points were collected only to understand the feasibility of data collection and not to conduct hypothesis testing. Key clinical data and their measurement are briefly summarised as follows (further details on scales used are provided in the online supplemental material):

10.1136/emermed-2024-213905.supp1 Supplementary data

#### Baseline

- Demographics, injury details, relevant medical history and Clinical Frailty Scale (CFS)^15^: collected by researcher from clinical notes.
- Retrospective pre-injury and baseline post-injury health EQ-5D-5L^16^: completed with participant/relative/carer.
- Timed Up and Go test.^17^

#### 72 hours postrandomisation (intervention period) collected until discharge if sooner

- Patient-reported pain scores: 4-hourly pain assessment using a Visual Analogue Scale (VAS) (scaled from 0 to 100). Recorded in a booklet provided to the patient.
- Frailty-specific outcomes: Abbey Pain Scale,^18^ 4-AT delirium assessment tool,^19^ constipation (Bristol Stool Chart), Timed Up and Go test.^17^ Obtained by researchers.
- Analgesia; ED and inpatient (72 hours) analgesic prescriptions, advanced analgesic provision (patient controlled analgesia (PCA), epidural, nerve block). Obtained by researchers from medical records.

#### 30 days (+10 days) postrandomisation

- Pulmonary complications: a priori proposed primary outcome for a definitive trial. Collected after review of medical records and adjudicated by site lead clinician.
- Delirium: binary measure of any inpatient episode of delirium recorded in clinical notes.
- Resource use: including admitted hospital length of stay, intensive care unit length of stay, unplanned readmission, discharge destination (notes review).
- Questionnaires: booklets containing EQ-5D-5L and ICECAP-O^16 20^ were sent by post to participants. Participants were permitted to complete these with the assistance of carers, although formal proxy versions of questionnaires were not provided.

### Sample size

As this was a feasibility trial, it was not appropriate to calculate a sample size to detect a specified treatment effect size. In line with published ‘rules-of-thumb’, we determined that a total sample size of 100 would be sufficient to provide estimates of feasibility measures (recruitment, retention, data completion and adherence).^21^ Recruitment was originally planned to take place over 18 months across three sites. However, trial set-up was delayed due to the COVID-19 pandemic. To achieve target recruitment within the funding period, the recruitment period was shortened to 12 months across seven sites.

### Statistical methods

Feasibility measures were analysed and reported following the Consolidated Standards of Reporting Trials guidance extension for feasibility studies to include descriptive and summary statistics both overall and by treatment arm.^22^

Descriptive statistics for participant characteristics and clinical outcome data were reported as means or medians with measures of dispersion for continuous outcomes and frequencies and percentages for categorical outcomes.

A priori thresholds for recruitment, follow-up and adherence were established to inform the feasibility of progression (table 2).

### Integrated qualitative study

Telephone interviews were undertaken with trial participants around 1 month (and up to 90 days) postrandomisation. Interviews and focus groups were conducted with clinicians/research staff closely involved in the trial set-up, recruitment and follow-up. These explored trial participation experiences including understanding and acceptability of processes, pain control including perceived benefits of lidocaine patches and views on trial outcomes (topic guides are included in the online supplemental material). Interviews and focus groups were audio-recorded, transcribed and analysed using thematic analysis.^23^ Qualitative findings were integrated with other elements using a ‘following a thread’ approach.^24^ This involved analysing each dataset and then using insights from the qualitative themes to contextualise and explain quantitative outcomes with data presented together.

### Health economic scoping

An evaluation of the feasibility of identifying and measuring health economics outcome data was completed, with the focus on establishing the most appropriate outcome measures for inclusion in a future economic evaluation alongside the definitive trial. The EQ-5D-5L (health-related quality of life) patient-reported questionnaire^16^ was completed at baseline, to capture retrospective pre-injury state and baseline post-injury state, and 30 days postrandomisation. In addition to the standard EQ-5D questionnaire, which typically elicits post-injury health status, we additionally assessed pre-injury status by making an approved change to the wording. The ICECAP-O (measure of capability in older people)^20^ was also collected at 30 days. Information on key resources, including length of stay, intensive care use and medication prescribing, was also collected.

## Results

**Figure 2:**
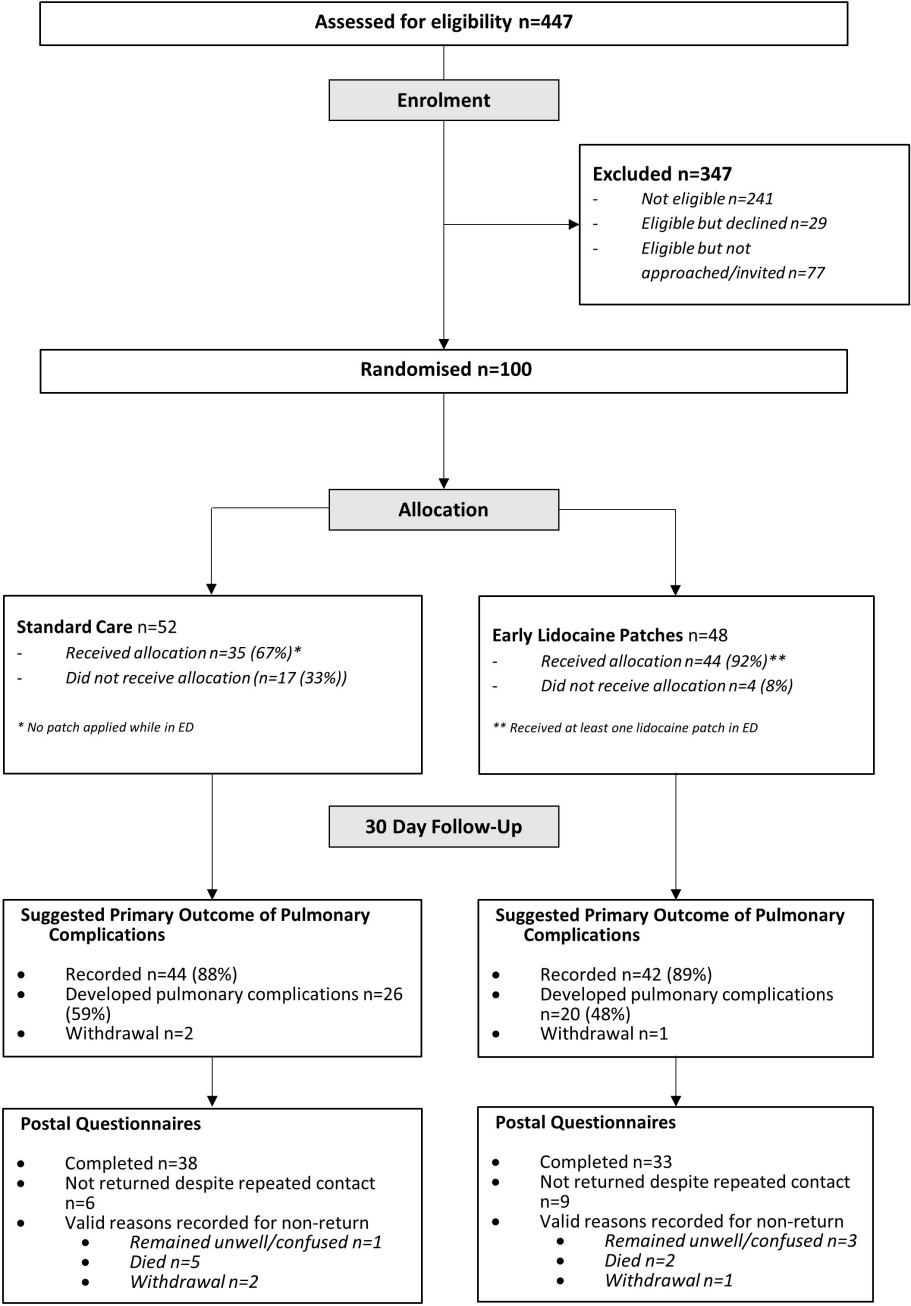
Screening, recruitment, allocation and follow-up (Consolidated Standards of Reporting Trials diagram).

Between 23 October 2021 and 7 October 2022, 447 patients were assessed for eligibility, of which 206 were eligible; of these, 29 declined and 77 were not approached. Therefore, 100 patients were randomised; 48 participants were allocated to lidocaine patches and 52 to standard care (figure 2). Six participants died prior to the 30-day follow-up timepoint and three participants withdrew from questionnaire completion, but had clinical data retained for analysis. Baseline characteristics were well balanced between groups (table 1).

**Table 1:** Baseline demographics and injury characteristics

N=denominator if data missing	Lidocaine patches N=48	Standard care N=52	All participants
Age, median (IQR), years	83 (74–88)	83 (75–86)	83 (74–88)
Sex			
Women (%)	23 (48)	24 (46)	47 (47)
Men (%)	25 (52)	28 (54)	53 (53)
Ethnic origin (%)			
White	45 (94)	47 (90)	92 (92)
Usual residence (%) n=99			
Own home	43 (90)	49 (96)	92 (92)
Care needs prior to admission if own home (%) n=88			
Independent	32 (78)	34 (72)	66 (75)
Informal family care	5 (12)	9 (19)	14 (16)
Package of care	4 (10)	4 (9)	8 (9)
Clinical Frailty Scale, median (IQR)	4 (3–5)	4 (3–5)	4 (3–5)
Relevant medical history (%)			
Chronic lung disease n=97	11 (23)	9 (18)	20 (21)
Current smoker n=91	7 (15)	4 (9)	11 (12)
Ex-smoker n=91	16 (35)	15 (33)	31 (34)
Pre-injury anticoagulants n=91	10 (22)	10 (22)	20 (22)
Previous diagnosis of cognitive impairment n=97	6 (13)	7 (14)	13 (13)
Mechanism of injury (%) n=99			
Fall from <2 m	35 (73)	45 (87)	80 (81)
Fall from >2 m	7 (15)	1 (2)	8 (8)
Road traffic accident	4 (8)	5 (10)	9 (9)
Pedestrian hit by vehicle	1 (2)	0	1 (1)
Crush injury	1 (2)	0	1 (1)
Prehospital treatment			
Time, mean (SD), hours between injury and arrival in hospital n=98	20 (27)	15 (19)	17 (23)
Mode of transport to hospital n=98			
Ambulance (%)	35 (73)	42 (84)	77 (79)
Private transport (%)	13 (27)	8 (16)	21 (21)
Analgesia			
Paracetamol (%) n=92	23 (53)	27 (55)	50 (54)
Morphine (%) n=93	11 (24)	17 (35)	28 (30)
Respiratory observations (first recorded in ED)			
RR, mean (SD), n=94	20 (3.1)	20 (3.3)	20 (3.2)
Percentage oxygen saturations, median (IQR), n=94	97 (95–98)	96 (94–99)	97 (95–98)
Percentage of oxygen administered (%), n=89			
Room air	18 (41)	12 (27)	30 (34)
22%–24%	20 (45)	28 (62)	48 (54)
>24%	6 (14)	5 (11)	11 (12)
Chest injury details			
Diagnosis through CT (%), n=93	43 (96)	42 (89)	85 (91)
Side of rib fracture(s) (%), n=97			
Right	27 (57)	20 (40)	47 (48)
Left	16 (34)	19 (38)	35 (36)
Bilateral	4 (9)	11 (22)	15 (16)
Total number of rib fractures, mean (SD), n=81	4 (2.1)	4 (2.7)	4 (2.0)
Displaced rib fracture (%), n=88	18 (38)	22 (46)	40 (45)
Flail chest—clinical (%), n=98	4 (9)	5 (10)	9 (9)
Flail chest—radiological (%), n=97	7 (15)	8 (16)	15 (16)
Haemothorax (%), n=97	5 (11)	7 (14)	12 (12)
Pneumothorax (%), n=97	10 (21)	13 (26)	23 (24)
Pulmonary contusion (%), n=95	0	4 (8)	4 (4)
Requirement for intercostal chest drain in ED (%), n=92	5 (11)	1 (2)	6 (7)
STUMBL score*, median (IQR), n=77	21 (18–26)	21 (16–33)	21 (16–33)
EQ-5D-5L			
EQ-Visual Analogue Scale (pre-injury†), median (IQR), n=83	80 (60–90)	80 (68–90)	80 (60–90)
EQ-Visual Analogue Scale (post-injury), n=81	50 (25–70)	50 (25–70)	50 (25–70)
Tariff (pre-injury baseline), median (IQR), n=86	0.84 (0.73–1)	0.83 (0.57–0.95)	0.84 (0.66–1)
Tariff (post-injury baseline), median (IQR), n=76	0.53 (0.24–0.66)	0.34 (0.26–0.60)	0.44 (0.25–0.63)

*The STUMBL score is a prognostic model comprising five risk factors: age, number of rib fractures, pre-existing chronic lung disease, use of pre-injury anticoagulants and oxygen saturation on initial assessment in ED. A median score of 21 equates to a 70% probability of developing adverse pulmonary complications.^3^

†Pre-injury status was collected through approved change of wording to the EQ-5D-5L: under each heading, please tick the ONE box that best describes your health BEFORE YOUR INJURY.

STUMBL, STUdy of the Management of BLunt chest wall trauma.

Participants were predominantly women (47%), of white British ethnicity (92%), with a median age of 83 years (IQR 74–88). Participants were predominantly admitted from their own homes (92%), were independent (75%) but were living with very mild frailty (median CFS 4; IQR 3–5). The most common mechanism of injury was a fall from <2 m (81%). On average, participants sustained four rib fractures (SD 2.0)and they were at high risk of developing pulmonary complications at baseline (median STUMBL score 21 (IQR 16–33)), equating to a 70% risk.^3^

### Feasibility outcomes

Table 2 details the prespecified progression criteria around recruitment, follow-up and adherence together with observed results.

**Table 2:** Prespecified progression criteria and observed results

Feasibility outcome	Prespecified progression criteria			Feasibility trial outcome	Caveats
	Green (progress)	Amber (amend)	Red (stop)		
Recruitment	**>70% expected recruitment**	50%–70% expected recruitment	<50% expected recruitment	>70% with 100 recruited in 12 months	Extended recruitment from three sites to seven, to mitigate delays from COVID-19 pandemic
Follow-up	**≥75% of data for suggested primary outcome of 30-day pulmonary complications**	65%–74% of data for suggested primary outcome of 30-day pulmonary complications	<65% of data for suggested primary outcome of 30-day pulmonary complications	≥75% with data complete for 86/97 (89%) participants after three withdrawals	Data incomplete for many frailty-specific secondary outcomes
Adherence	**≥75% adherence to the intervention**	65%–74% adherence to the intervention	<65% adherence to the intervention	Overall adherence*: 77/97 received allocated treatment (79%)	Significant cross-over in standard care arm with 33% non-adherence

Bold indicates progression level met (green, amber, red) for each outcome.

*Adherence was defined as receiving at least one lidocaine patch in the ED (intervention) or no lidocaine patch in ED (standard care).

#### Recruitment and consent

An average of 14 participants were recruited per site (range 3–37) in 12 months. Participants were predominately recruited from major trauma centres (n=87).

Agreement to participate was largely obtained from patients (70%): personal consultees (in England) or legal representatives (in Scotland) were approached in 27% of cases, and professional consultees were used in 3% of cases.

In the qualitative research, clinical and research staff closely involved in delivering the trial reported challenges in recruiting within the ED setting. These challenges included general ED pressures, reliance on referrals from wider clinical teams not directly engaged in the research, resource-intensive monitoring of ED attendances for potentially eligible patients, the necessity to rapidly attend ED (when not based in the department) to approach patients and lack of out-of-hours research staff (although some engaged clinicians were able to recruit out of hours). However, they were able to recruit well by raising awareness of the trial and fostering good collaborative relationships with the wider ED clinical team members, who were able to actively participate in patient identification. Insights from older patients were limited due to challenges with interview engagement (of 26 participants approached for interviews, 7 took part, 5 declined, 14 did not respond). However, older patients interviewed welcomed being approached and were willing to participate in the trial because they wanted to help, but were sometimes unsure of trial details. Staff needed to consider older patients’ vulnerability, and carefully manage consent processes to avoid overwhelming them, while ensuring their full understanding of involvement and the option not to participate.

#### Follow-up and data completeness

The proposed primary outcome of adverse pulmonary complications at 30 days was completed for 86% of participants (data missing in 14%, due to transfer to remote facilities or discharge home and no further records were available). For the 30-day patient-completed questionnaires, in total 71 were returned (fully or partially completed), 15 were unreturned despite repeated contact and 14 had reasons recorded for non-return (7 deaths, 4 remained unwell/confused, 3 withdrawals). This equates to an overall return rate of 71% but rising to 83% when return was anticipated. Qualitative findings regarding questionnaire completion highlighted the unblinded nature of the intervention, with standard care participants not feeling part of the trial, potentially impacting their understanding of completing questionnaires in future research.

Pain and frailty-specific outcomes (important secondary outcomes but not included in prespecified progression criteria) were not feasible to collect as completeness was <65%. Table 3 summarises data completeness on these measures and qualitative exploration of factors influencing data collection.

**Table 3:** Pain and frailty-specific outcomes that were not feasible to collect and qualitative exploration of factors influencing data collection

Outcome	Method of data collection	Completeness*	Qualitative† findings
Patient-reported pain scores	Paper diary completed at 4 hourly intervals (excluding sleep) as inpatient for 72 hours with support from a researcher where available	22%	Staff encountered difficulties as some participants were non-compliant and lacked a clear understanding of the assessment process, leading to challenges in obtaining meaningful responses. These issues were believed to be specific to the older patient cohort. “We had awful trouble with the pain score side of it with the paperwork. Some patients were just completely non-compliant with doing the pain scores. Of those ones that we did help, or try and assist to do the pain scores some didn’t really get what we were asking them if that makes sense? I think it was difficult to gauge”. (Research nurse, focus group 2) “Not everyone identifies the level of or acknowledges the pain… particularly the elderly don’t want to be a bother… I think that’s more a systemic issue rather than specific to the trial, but I think it’s the reality of this patient group and this generation who are the ones who don’t want to be a burden on people”. (Clinician, focus group 2) “They really want to get it right; they really want to tell you the right answer… Like 95 out of 100, even though you’ve got seven fractured ribs, giving it their best shot at pleasing you, because they think you want… So, it’s even then it’s not ideal I don’t think with someone in that amount of pain”. (Clinician, focus group 1)
Abbey Pain Scale	Completed daily by research team/transcribed from clinical notes where routinely collected	63%	Clinicians found the observable pain assessment tool preferable to self-reported assessment, but the tool may be unfamiliar to ward nurses. “Using other pain tools like the Abbey Pain Tool could be useful, so that’s a pain tool you don’t have to ask the patient, they can look for things like grimacing, or behaviour changes in order to deliver medications. I don’t think many of the nurses know about that tool, some of them on the frailty units, but outside of that probably not”. (Clinician, focus group 1)
4-AT (delirium)	Completed daily by research team/transcribed from clinical notes where routinely collected	59%	Difficulty and inconsistency in completing delirium scores due to lack of a routine collection in practice, limited familiarity with patients, and variation in interpretation of questionnaire responses among clinicians. “The actual questionnaires like the delirium score and the… it’s not an easy score to complete, is it? No. Or to interpret some of the questions. We’ve all struggled, haven’t we?” (Clinicians exchange, focus group 1) “We don’t have an official delirium pathway in this hospital, so many of us are doing delirium scores, they’re happening haphazard”. (Clinician, focus group 2) “We’re research nurses but we’re not giving nursing care to these patients every day for hours and hours. So, we know them for that short period that we recruit them, and then we might pop in to do the questionnaires and that’s it. We don’t know them as nurses would on the ward who looked after them every day, and it’s even more difficult for that reason… Our opinions about whether they’re eccentric or delirious might vary”. (Research nurse, focus group 2)
Bristol Stool Chart (constipation)	Notes review by researcher at 72 hours (routinely collected at all sites)	45%‡	Completeness might stem from broader issues such as ward workload, patient access and transfers. “Ward workload, they’re just so short-staffed they cannot do anything other than the essentials at the moment”. (Clinician, interview 1) “I think the usual things when you are doing the questionnaires, either you find them sleeping, or they’re doing an intervention, or they went for another SA catheter, and so those are a bit sometimes missed”. (Clinician, focus group 2) “They would come in under major trauma, and then they were often divvied out to different wards… so very quickly it can be decided it’s long-term care issues, and long stay care of the elderly ward. So, they’re trying to bring these… the paper form with them to the different wards, and then you’re going to try and have to get another group of nurses on board to do this”. (Research nurse, interview 1)
Timed Up and Go test (immobility)	Performed by researcher at baseline (in ED) and at 72 hours	6% participants completed at baseline 15% completed at 72 hours	Overwhelming patients with research procedures. “We’re not the only team that wants to see them….They’ve got physiotherapy, they’ve got OT, they might have people coming to assess them for rails at home, and it goes on and on, and then there are relatives coming to visit them. So actually, a lot of the time we’ve gone and they’re just exhausted of being asked questions, and poked and prodded”. (Research nurse, focus group 1)

*Where applicable, data collection was attempted during the 72-hour intervention period, and completeness represents a summary average over this time period.

†Sixteen clinicians from three sites took part in two focus groups (n=13) (one face-to-face and one online) and online interviews (n=3). Clinicians encompassed various professions such as nursing (including research nurses and those involved in acute care/frailty care), pharmacy, anaesthesiology, emergency medicine and geriatrics. Six participants (mean age 78 years) from both trial arms (control n=3, intervention n=3) and one carer were interviewed.

‡90 participants remained in the hospital at 72 hours. Of these, 50 did not have bowel movements during that time period. Therefore, a maximum of 40 participants could have had their Bristol Stool Chart recorded, of which 18 were documented.

#### Adherence

In the intervention arm, 44/48 (92%) participants had at least one lidocaine patch applied in ED at a median time of 393.5 min after arrival. In the standard care arm, 17/52 (33%) participants also had a lidocaine patch applied in ED and were therefore classed as non-adherent. However, overall adherence was 79% meeting the prespecified green criteria for feasibility (>75%). Themes identified in the qualitative research with clinical/research staff addressing variation in care included standard care (some hospitals use patches as standard care, others do not), patch application (eg, where best to place patches in the presence of multiple fractures), provision of nerve blockade (the ongoing use of lidocaine patches when nerve blocks are subsequently used), equipoise (mixed views on the benefits of patches) and patch acceptability (perceived benefits of patches to patients) (see online supplemental material for details).

### Clinical outcomes

#### 72-hour outcomes

Data on ED and inpatient (72 hours) analgesic prescriptions, together with advanced analgesic provision (PCA, epidural, nerve blocks) were collected in >75% of participants (table 4) Analgesic prescriptions within ED and as an inpatient were similar between arms. Overall, 33/97 (34%) participants had advanced analgesia with 21/97 (22%) receiving some form of nerve blockade and 12/97 (13%) receiving PCA within the 72-hour intervention period.

**Table 4:** Clinical outcomes

N=denominator if data missing	Lidocaine patches N=47	Standard care N=50	All participants N=97
72-hour outcomes			
Analgesic prescription in ED, median number of prescriptions (IQR), n=93			
Paracetamol	1 (0–1)	1 (0.5–1)	1 (0–1)
Weak opioid	0	0	0
Strong opioid	1 (0–2)	1 (0–1)	1 (0–2)
Other non-opioid analgesia	0	0	0
Analgesic prescription as inpatient in 72 hours, median number of prescriptions (IQR), n=86			
Paracetamol	3 (3–6)	4 (3–6)	4 (3–6)
Weak opioid	0 (0–2)	0 (0–1)	0 (0–1)
Strong opioid	4 (1-6)	4 (2-7)	4 (2-6)
Other non-opioid analgesia	0 (0–1)	0 (0–1)	0 (0–1)
Advanced analgesia within 72 hours of admission, (%), n=97			
Patient controlled analgesia	4 (9)	8 (16)	12 (12)
Thoracic epidural	2 (4)	1 (2)	3 (3)
Intercostal nerve block/catheter	2 (4)	0	2 (2)
Serratus anterior block/catheter	2 (4)	4 (8)	6 (6)
Erector spinae plane block/catheter	4 (9)	6 (12)	10 (10)
30-day outcomes			
All pulmonary complications* (%), n=86	20 (48)	26 (59)	46 (53)
Specific pulmonary complications included in composite outcome (%)			
Type 1 respiratory failure, n=91	11 (24)	16 (35)	27 (30)
Type 2 respiratory failure, n=90	1 (2)	1 (2)	2 (2)
Lower respiratory tract infection (non-pneumonia),† n=89	6 (14)	8 (18)	14 (16)
Pneumonia,† n=89	7 (16)	6 (13)	13 (15)
New pleural effusion (>24 hours after injury), n=91	5 (11)	6 (13)	11 (12)
Ventilator-assisted pneumonia, n=91	0	0	0
Adult respiratory distress syndrome, n=91	0	0	0
Empyema, n=91	0	0	0
Pulmonary embolism, n=90	0	0	0
COVID-19 pneumonitis, n=91	1 (2)	1 (2)	2 (2)
Other respiratory complication, n=85	1 (2)	3 (7)	4 (5)
In-hospital mortality† (%), n=87	0	2 (5)	2 (2)
Development of delirium (%), n=90	5 (11)	7 (15)	12 (13)
Unplanned hospital re-admission (%), n=79	4 (10)	2 (5)	6 (8)
Admitted to intensive care (%), n=87	4 (10)	3 (7)	7 (8)
Total length of hospital stay, median days (IQR), n=80	11.4 (6.3–18.5)	6.9 (4.4–11.1)	9.1 (5.2–15.4)
Discharge destination (%), n=76			
Home/Usual residence where residential or nursing care (return to baseline)	25 (62)	26 (68)	51 (67)
Home with increased package of care	6 (15)	3 (8)	9 (12)
Residential or nursing care (where admitted from own home)	3 (8)	0 (0)	3 (4)
Rehabilitation unit	3 (8)	5 (14)	8 (11)
Other (including repatriation to local hospital)	2 (5)	3 (8)	5 (7)
Health economic scoping (day 30 returned questionnaires)			
EQ-Visual Analogue Scale, median (IQR), n=69	70 (50–80)	70 (50–80)	70 (50–80)
EQ-5D-5L tariff, median (IQR), n=44	0.61 (0.22–0.69)	0.57 (0.28–0.78)	0.59 (0.27–0.74)
ICECAP-O tariff, median (IQR), n=65	0.84 (0.65–0.90)	0.77 (0.65–0.89)	0.77 (0.65–0.89)

*Proposed primary outcome for a definitive trial: binary measure of pulmonary complications—NO complications vs ANY complication(s). Pulmonary complications collected were: type 1 respiratory failure (PaO_2_ <8 kPa (60 mm Hg) on any ABG and/or new oxygen requirement), type 2 respiratory failure (PaCO_2_ >6 kPa (50 mm Hg) on any ABG and/or consideration/administration of non-invasive ventilation), pulmonary embolism, pneumonia (confirmed airway opacification on imaging >24 hours post-injury and a prescription of antibiotic), ventilator-associated pneumonia, adult respiratory distress syndrome, lower respiratory tract infection (prescription of antibiotic in the absence of airway opacification on imaging), new pleural effusion >24 hours after injury, empyema, COVID-19 pneumonitis and other respiratory complication. Adapted with the addition of COVID-19.^3^

†In-hospital mortality was not included within the suggested primary outcome of pulmonary complications, however the trial participants who died had met the pulmonary complications outcome prior to death.

#### 30-day outcomes

Overall, 46/86 (53%) participants with complete data met the outcome of composite pulmonary complications within 30 days; 20 (48%) in the lidocaine patch arm and 26 (59%) in the standard care arm. The median length of hospital stay was 9.1 days (IQR 5.2–15.4) and over 30% of participants did not return to their baseline level of function on discharge (requiring increased package of care, residential, nursing or rehabilitation). Descriptive data on all 30-day outcomes is included in table 4.

### Health economic scoping

We achieved our objectives in terms of piloting instruments of data collection: administration of EQ-5D-5L and ICECAP-O measures and case report forms to record length of stay, use of analgesia and discharge destination (table 4).

As anticipated EQ-VAS at baseline (measuring overall health status with 100 being best imaginable health) were reported as higher pre-injury (median 80 (60–90)) compared with post-injury (median 50 (25–70)). At 30 days, EQ-5D-5L completeness was 44% and ICECAP-O was 65%. In terms of the trajectory of health status, as anticipated the baseline EQ-5D-5L post-injury tariff had the lowest median (0.44 (0.25–0.63)) while at 30 days these data indicated participants had only partially recovered in terms of health status (0.59 (0.27–0.74)) (table 4). The overall median ICECAP-O tariff at 30 days was 0.77, which is slightly below a published population norm of 0.81.^25^

## Discussion

This trial suggests it is feasible to recruit older patients with rib fracture(s) in an emergency setting. Consent processes modified for older patients were effective and acceptable to patients and carers. However, pain and frailty-specific outcomes were not feasible to collect. While these were not anticipated primary outcomes for a future trial, they are clearly important secondary outcomes in this population. Our qualitative work highlighted areas for improvement in this regard. These include bespoke training for researchers when unfamiliar with measures (Abbey Pain Scale, 4-AT delirium assessment tool), embedding measures such as 4-AT delirium assessment tool into clinical practice and increased recognition of the potential to overwhelm older injured patients through research procedures when designing trials. It should be noted that the World Hip Trauma Evaluation platform study appears to have overcome many of these barriers to data collection in a similar population.^26^

Data collection for the suggested primary outcome of a definitive trial (adverse pulmonary complications) was feasible, and the high rates of this outcome within the population confirm that it remains a target outcome for early analgesic interventions in older patients with rib fracture(s).

Paper-based, mailed out, patient-completed questionnaires were returned at high rates, suggesting that this remains an acceptable option for older participants in research. This aligns with consensus recommendations that alternatives should be offered to digital data collection to avoid digital exclusion in older patients.^12^ However, for those patients with cognitive impairment, consideration of formal proxy versions of questionnaires should be considered where available.

While adherence to the intervention was high and overall adherence was deemed feasible, significant crossover in the standard care arm was seen. This finding suggests clinicians may lack equipoise in sites where lidocaine patches are already in use; this was confirmed in our healthcare professional focus groups. However, these focus groups also highlighted discrepancies in prescribing/availability and a recognition of the potential harm of overuse of lidocaine patches (at the expense of other analgesic modalities). In order to overcome these challenges in equipoise, avoid crossover and fully understand the clinical effectiveness of topical lidocaine, a definitive trial would need to test active patches against placebo patches rather than standard care.

In this trial, older patients admitted to hospital with radiologically confirmed rib fracture(s) were living with very mild frailty (median CFS 4) and were predominantly injured after a fall from standing (<2 m), a finding consistent with previous reports.^27^ Despite having isolated rib fracture(s), many participants had prolonged hospital stays (median 9 days) and >30% did not return to baseline functional status on discharge. STUMBL scores recorded at baseline suggested a population at high risk of developing adverse pulmonary complications and this finding was confirmed in 30-day outcome collection. Development of delirium appeared lower than reported in other cohorts,^6^ but may reflect a lack of robust data collection. Notable findings that may provide targets for service improvements include prolonged times between injury and hospital arrival (20 hours) and low rates of prehospital analgesia administration. In addition, in-hospital (72 hours) analgesic prescriptions appear to rely heavily on strong opioid analgesia, with more advanced analgesic modalities being used in only around one-fifth of this vulnerable patient group.

Rib fracture(s) were diagnosed by CT in over 90% of cases. This may reflect a more liberal use of CT in older patients with suspected trauma following influential reports such as Trauma Audit Research Network Major Trauma in Older People^28^ and the majority of sites being major trauma centres. However, this finding may also reflect selection bias towards more severely injured patients, given that our inclusion criteria required radiological confirmation of rib fracture(s) and prior studies have demonstrated a poor sensitivity of X-ray diagnosis, with only 40% accuracy in older patients.^29^ Amending the inclusion criteria to include patients with clinically suspected (rather than radiologically confirmed) rib fractures may mitigate against this selection bias and also allow the inclusion of those patients who are less severely injured and potentially more frail.

Our health economic scoping revealed key findings to be considered in future research involving older adults in emergency settings. Modification of the standard EQ-5D to obtain retrospective pre-injury health status may be beneficial in assessing specific impacts of injury in economic modelling. However, since response rates to the ICECAP-O were higher than for the EQ-5D at 30 days, which may reflect a patient preference for completing a measure specifically designed for use in older people, it is possible that this is a more appropriate measure for use in a definitive trial.

## Conclusions

This trial has demonstrated that recruitment of older patients with rib fracture(s) in an emergency setting for the evaluation of early analgesic interventions (in the form of lidocaine patches) is feasible. Refinement of data collection, with a focus on collecting pain and frailty-specific outcomes, as well as intervention delivery, is needed before progressing to a definitive trial.

## Data Availability

Data are available on reasonable request. Further information and patient-facing materials (including model consent forms) are available at https://relief.blogs.bristol.ac.uk/. Data available on request.
