# Supplementary material for: The RELIEF feasibility trial: topical lidocaine patches in older adults with rib fractures
Source: Emerg Med J. 2024 May 16;41(9):522–31. doi: 10.1136/emermed-2024-213905 (PMC11347219; doi:10.1136/emermed-2024-213905)
Supplement: Supplementary data [file emermed-2024-213905supp001.pdf]

Randomised Evaluation of topical Lidocaine patches In Elderly patients admitted to hospital with rib Fractures (RELIEF); the results of a feasibility trial and integrated qualitative study.

Supplementary Data

Contents

Appendix 1. Detailed description of scales used ... 2
Appendix 2. Topic Guides ... 7
Appendix 3. Factors influencing adherence ... 11

Clout M, *et al. Emerg Med J* 2024; 41:522–531. doi: 10.1136/emermed-2024-213905

Appendix 1 – Detailed description of scales used

Clinical Frailty Scale

The Clinical Frailty Scale is a way to summarize the overall level of fitness or frailty of an older adult after they have been evaluated by an experienced clinician. The score has been widely taken up as a judgement-based tool to screen for frailty and to broadly stratify degrees of fitness and frailty. It is not a questionnaire, but a way to summarise information from a clinical encounter with an older person, in a context in which it is useful to screen for and roughly quantify an individual's overall health status.

1 Very fit – robust, active, energetic, well motivated and fit; these people commonly exercise regulatory and are in the most fit group for their age

2 Well – without active disease, but less fit than people in category 1

3 Well, with treated comorbid disease – disease symptoms are well controlled compared with those in category 4

4 Apparently vulnerable – although not frankly dependent, these people commonly complain of being "slowed up" or have disease symptoms

5 Mildly frail – with limited dependent on others for instrumental activities of daily living

6 Moderately frail – help is needed with both instrumental and non-instrumental activities of daily living

7 Severely frail – completely dependent on others for the activities of daily living, or terminally ill

Of note Version 1 was used in the trial, this has been superseded by Version 2.

Reference:

A global clinical measure of fitness and frailty in elderly people, Rockwood et al, CMAJ . 2005 Aug 30;173(5):489-95. doi: 10.1503/cmaj.050051.

Timed up and go test

The Timed Up and Go (TUG) is a mobility test for frail older persons. The test consists of the time it takes for the patient to rise from an armed chair, walk 3 metres at their usual speed and with their usual gait aid, turn and return to the seated position in the chair. TUG is recommended as part of a safety assessment in older patients considered for discharge from the ED following a fall.

References:

Eagles D, Perry JJ, Sirois MJ, et al. Timed Up and Go predicts functional decline in older patients presenting to the emergency department following minor trauma†. Age Ageing. 2017;46(2):214-8.

Podsiadlo D, Richardson S. The timed "Up & Go": a test of basic functional mobility for frail elderly persons. J Am Geriatr Soc 1991; 39: 142–8.

4-hourly pain assessment: Visual Analogue Scale (VAS)

The Visual Analogue Scale (VAS) is used to measure the severity of pain. The VAS consists of a 100mm unmarked line with standardised wording: 'no pain' on the left of the line, and 'worst pain imaginable' on the right—the patient then places a mark on the line corresponding to their level of pain. For patients who have mild/moderate cognitive impairment the VAS was completed with the assistance of a researcher/member of the clinical team.

DAY 2 – MEASURE 1 *(EXAMPLE)*

1) Please mark a spot or X on the line below to show your pain intensity.

Score calculated by researcher (ONLY)

Score

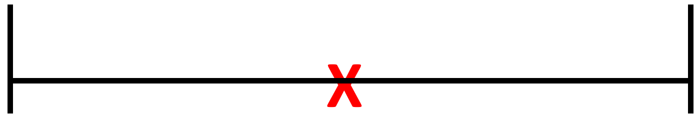

NO PAIN **PAIN AS BAD AS IT COULD BE**

2) Please state what 4-hour period of time this measure refers to. *(e.g. 08:00-12:00. If not using the 24-hour clock, please indicate whether AM or PM).*

7:30-11:30 (slept from 10pm through 'till 7:30am)

AM / ~~PM~~

Abbey Pain Scale

The Abbey Pain Scale is an instrument designed to assist in the assessment of pain in residents who are unable to clearly articulate their needs. The Scale does not differentiate between distress and pain, so measuring the effectiveness of pain-relieving interventions is essential.

The Abbey Pain Scale be used as a movement-based assessment. The staff recording the scale should therefore observe the patient while they are being moved, eg during pressure area care, while showering etc and complete the scale immediately following the procedure.

Clout M, *et al. Emerg Med J* 2024; 41:522–531. doi: 10.1136/emermed-2024-213905

DAY X (Abbey Pain Score): **How to use scale:** While observing the patient, score questions 1 to 6.

- **If it was not feasible to record/complete this assessment, please record this below (or mark N/A):**

Please provide reason(s) why not feasible *(e.g. patient unavailable, patient asleep, weekend, no research staff available).* N/A ☐

	D	D	-	M	M	-	Y	Y	Y	Y		H	H	:	M	M
Day 1 Date:			-			-					Time:			:		

		H	H	:	M	M
**Latest pain relief given was:**	______ at:			:		

Q1. Vocalisation e.g. whimpering, groaning, crying
(Absent 0 Mild 1 Moderate 2 Severe 3) ☐

Q2. Facial expression e.g. looking tense, frowning, grimacing, looking frightened
(Absent 0 Mild 1 Moderate 2 Severe 3) ☐

Q3. Change in body language e.g. fidgeting, rocking, guarding part of body, withdrawn
(Absent 0 Mild 1 Moderate 2 Severe 3) ☐

Q4. Behavioural Change e.g. increased confusion, refusing to eat, alteration in usual patterns *(Absent 0 Mild 1 Moderate 2 Severe 3)* ☐

Q5. Physiological change e.g. temperature, pulse or blood pressure outside normal limits, perspiring, flushing or pallor *(Absent 0 Mild 1 Moderate 2 Severe 3)* ☐

Q6. Physical changes e.g. skin tears, pressure areas, arthritis, contractures, previous injuries *(Absent 0 Mild 1 Moderate 2 Severe 3)* ☐

Jennifer Abbey, Neil Piller, AnitaDe Bellis, Adrian Esterman, Deborah Parker, Lynne; Giles and Belinda Lowcay (2004) The Abbey pain scale: a 1-minute numerical indicator for people with end-stage dementia, International Journal of Palliative Nursing, Vol 10, No 1pp 6-13. (This document may be reproduced with this acknowledgement retained)

4

4-AT delirium assessment tool (version 1.2, www.the4AT.com)

The 4AT is a screening instrument designed for rapid initial assessment of delirium and cognitive impairment.

4-AT **A. DAY X performed:**	**i.** Date:	D D - M M - Y Y Y Y	**ii.** Time:	H H : M M
		☐☐ - ☐☐ - ☐☐☐☐		☐☐ : ☐☐

- **If it was not feasible to record/complete this assessment, please record this below (or mark N/A):**

Please provide reason(s) why not feasible *(e.g. patient unavailable, patient asleep, weekend, no research staff available).* N/A ☐

1. ALERTNESS

This includes patients who may be markedly drowsy (eg. Difficult to rouse and/or obviously sleepy during assessment) or agitated/hyperactive. Observe the patient. If asleep, attempt to wake with speech or gentle touch on shoulder. Ask the patient to state their name and address to assist rating. *Tick one answer*

	Score	Tick
Normal (fully alert, but not agitated, throughout assessment)	**0**	☐
Mild sleepiness for <10 seconds after waking, then normal	**0**	☐
Clearly abnormal	**4**	☐

2. AMT4

Age, date of birth, place (name of the hospital or building), current year. *Tick one answer*

No mistakes	**0**	☐
1 mistake	**1**	☐
2 or more mistakes/untestable	**2**	☐

3. ATTENTION

Ask the patient: *"Please tell me the months of the year in backwards order, starting at December."* To assist initial understanding one prompt of *"what is the month before December?"* is permitted.

Months of the year backwards *Tick one answer*

Achieves 7 months or more correctly	**0**	☐
Starts but scores <7 months / refuses to start	**1**	☐
Untestable (cannot start because unwell, drowsy, inattentive)	**2**	☐

4. ACUTE CHANGE OR FLUCTUATING COURSE

Evidence of significant change or fluctuation in: alertness, cognition, other mental function (eg. Paranoia, hallucinations)

arising over the last 2 weeks and still evident in last 24hrs.	*Tick one answer*	
No	**0**	☐
Yes	**4**	☐

Reference:

Saller T, MacLullich AMJ, Perneczky R. The 4AT - an instrument for delirium detection for older patients in the post-anaesthesia care unit. Anaesthesia. 2020;75(3):410.

Bristol Stool Chart

The Bristol Stool Chart is a medical aid designed to classify stools into seven groups. it can be used to identify whether patients may be experiencing constipation.

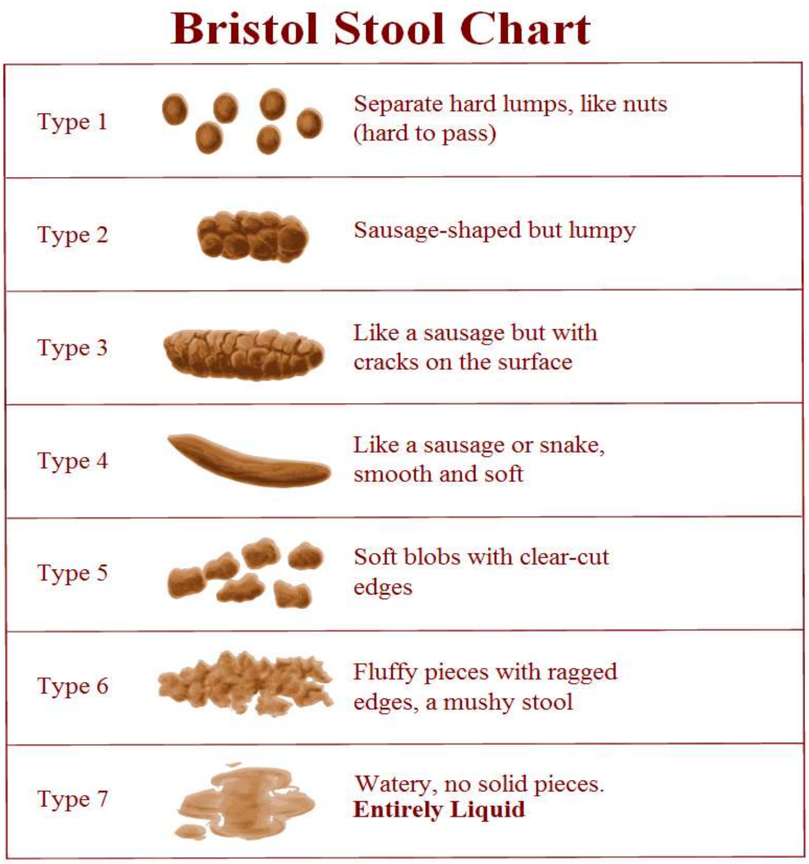

Reference:

Lewis SJ, Heaton KW (1997) Stool form scale as a useful guide to intestinal transit time. Scandinavian Journal of Gastroenterology 32: 920-4.

Appendix 2. Topic Guides

TOPIC GUIDE FOR QUALITATIVE INTERVIEWS PATIENTS AND PEOPLE WITH CARER RESPONSIBILITIES

1. Introduction and background:

Thanks, introduce self, and check who you (the interviewer) are speaking to. Once satisfied that speaking with appropriate person(s); re-state purpose of the interview. Confirmation that interview will be recorded, right to withdrawal, issues of confidentiality, anonymisation and informed consent, expected length of interview. If agree, start audio recorder.

Verbal consent

- Do you agree to our conversation being audio recorded?
- You are free to stop the interview at any point and you may skip questions you would prefer not to answer?
- You understand that quotations from the interview may be used to illustrate our findings but it will not be possible to trace who said them?
- You understand that we will keep a written record of the interview but without anything that could identify you for future research?

Background information on participant

Patient/Person carer responsibilities, which hospital attended.

2. Topics to be covered:

i. Experiences of their participation in the trial

- Please describe what happened when you were admitted following your rib injury?
- Randomisation: How did you feel about being allocated to one of two groups (randomisation)? Was this explained to you well enough? Did you have any questions?
- Informed consent:
  - Can you remember how you heard about the study?
    - who spoke to you about the study? What did they say? If seen by more than one member of the A&E team, was the information given consistent?
- How did you find the study information? Was it enough of an explanation of the study and what was involved? Did they understand what they were being asked to do? Is there anything that you didn’t understand/wanted more information on? Was the information given in an understandable language (considering ethnicity/communication difficulties)? Were you happy with the level of privacy you were given to discuss the study – was dignity (culturally, age etc) preserved?
  - Do you remember giving written consent? Was anyone with you?

- Follow-up questionnaires: Did the staff explain about the questionnaires to complete during your follow-up? What was your understanding about what you needed to do?
- Is there anything that we could improve about the study?
- Do you think it would be acceptable to use a fake/dummy patch in future research?
- Are you glad you took part in the study? Why, why not? Would you take part in the study again? Would you recommend family/friends to take part in the study? Explore.

ii. Pain control

- Was your pain controlled well enough during your admission?
- Intervention group-do you think lidocaine patches improved your pain control? Was 72 hours long enough for them to work?
- Control group: would you liked to have had additional pain control e.g. patches?
- Contamination (where patches applied later outside of trial processes): would you have liked patches to have been applied earlier during your admission?
- Following discharge: did you have any ongoing issues related to pain from your rib injury?

iii. Outcome measures

- We are looking to see if lidocaine patches reduce the chances of developing a chest infection. What do you feel is an important thing(s) to ask patients about (measure)? What else would be important for us to measure?
- Are there any other effects of injury or side effects of medications following rib fractures we should explore with patients?

iv. **<u>For people with carer responsibilities only</u>**

- Were trial processes easy to follow for patients with cognitive impairment/dementia?

NB: This is a guide only. Interviewer to use further prompts and probes in response to patients' responses. Interviewer to use own discretion to omit questions or alter wording as appropriate during interviews.

Clout M, *et al. Emerg Med J* 2024; 41:522–531. doi: 10.1136/emermed-2024-213905

TOPIC GUIDE FOR HEALTHCARE PROFESSIONALS (HCPs) FOCUS GROUPS.

1. Introduction and background.

To be conducted face-to-face or via telephone/video conferencing. Thanks, introduce self, state purpose of focus group. Confirmation that interview will be recorded, right to withdrawal, issues of confidentiality, anonymisation and informed consent, expected length of interview. – if agree start audio recorder.

Written consent

Background information on participants – HCP role, involvement in trial.

2. Topics to be covered:

v. Experiences of their participation in the trial

- Please describe your experiences of trial processes.
- Randomisation: How did you feel about the randomisation process? Was it appropriate to randomise patients in the ED? Explore equipoise around intervention
- Informed consent: Was the consent process adequate/too detailed/easy to undertake in the ED?
- Follow-up questionnaires: Were follow-up questionnaires to long/too short and easy to complete. How can they be improved??
- Is there anything that we could improve about the study?
- Do you think it would be acceptable to use a placebo patch in future research?
- What are the barriers/facilitators to using lidocaine patches in routine practice?
- Were inclusion/exclusion criteria applicable to routine practice? Should patients being discharged from ED be recruited in future trials?

vi. Pain control

- What are the challenges in delivering pain control to older patients with rib fractures?
- Did participants who had lidocaine patches report improvements in pain control?
- What are the barriers/facilitators to improved pain control in older patients with rib fractures?

vii. Outcome measures

Clout M, *et al. Emerg Med J* 2024; 41:522–531. doi: 10.1136/emermed-2024-213905

- What are the most important outcome measures for a trials in rib fracture pain control?
- Are there any other effects of injury or side effects of medications following rib fractures we should explore with patients?

viii. Participants with cognitive impairment

- Were trial processes easy to follow for patients with cognitive impairment/dementia?

NB: This is a guide only. Interviewer to use further prompts and probes in response to patients' responses. Interviewer to use own discretion to omit questions or alter wording as appropriate during focus groups.

Clout M, *et al. Emerg Med J* 2024; 41:522–531. doi: 10.1136/emermed-2024-213905

Appendix 3. Factors influencing adherence

**Theme**	**Participant quotes**
Variation in standard care pathways.<br>- Some hospitals already use lidocaine patches as standard treatment for rib fractures, while others do not.	*"It was either patch or standard and our standard they would still get the patch, so, either way, they would get a patch." (Clinician, interview 2)*<br><br>*"So that's [use of lidocaine patches] part of our protocol management for fractured ribs." (Clinician, focus group 2)*<br><br>*"The doctor would want the lidocaine patch, randomised for it or not, he would still want it because that's our policy." (Clinician, focus group 2)*<br><br>*"So that's not what would be prescribed here at all [lidocaine patches], so we would not have had that." (Clinician, focus group 2)*
Variation in access.<br>- Access to lidocaine patches varies between hospitals, with some having them readily available and approved, while others struggle to obtain them.<br><br>- Issues with prescribing patches.	*"It's not part of our standard formulary for the… certainly acutely we've never put a lidocaine patch on, because we can't, we don't even stock them, and certainly we've had a different experience obviously to [hospital] there, just as to what's available." (Clinician, focus group 2)*<br><br>*"We have access to lidocaine patches outside the trial. We prescribe them a lot." (Clinician, focus group 1)*<br><br>*"They are not routine stock for pharmacy. We have tried to get them as routine stock for some of the wards that we work on, but they're often out." (Clinician, focus group 1)*<br><br>*"I've had issues getting them prescribed, that's very hit and miss." (Clinician, focus group 1)*<br><br>*"I think the only difficulty that we came across with the electronic prescribing is normally because it's 24/7 A&E is open, you might have a patient coming in and they normally try to restrict prescriptions to daily in the morning or daily in the afternoon, or daily in the evening." (Clinician, interview 2)*
Lidocaine patch application issues<br>- Uncertainty about the correct application of patches	*"Timing, so the patients were often consented at out of hours, and so if it needs to go on and then come off 12 hours later it got very confusing about what to do next. So if someone had it put on at ten at night then it was coming off at ten in the morning, and so the patient was awake all day without their*

- Discrepancies in patch application<br><br>- Patient movement (e.g., wards and discharge)	*additional pain relief from the patch." (Clinician, focus group 1)*<br><br>*"I think a lot of issues did come up about the patches not being removed at the right time or taken off at the same time when they then moved from ED to a ward area." (Clinician, interview 3)*<br><br>*"It's prescribed for eight, but it doesn't always get put on." (Clinician, focus group 2)*<br><br>*"I was cutting one in half and using it, because I was putting one on the front, one on the back, and the same on the other side." (Patient 6, interview)*<br><br>*"We'll put a patch on, and then if it's a daily prescription it is quite difficult then to co-ordinate when the next dose or next administration should be… a huge barrier for… as part of the transfer of care between ED and an inpatient area." (Clinician, interview 3)*<br><br>*"We knew that the nurses on the ward were not going to be able to keep this up in the way that we would want over the three-day course. So that's why we made the decision for us to be the ones to apply it…. To be honest, a lot of the ward nurses aren't interested in anything other than the essentials of what they need to give. So, the trial drug to them was quite I think something that they just didn't want to have to deal with." (Clinician, interview 1)*
Patch acceptability (perceived benefits of patches to patients) Patients believed the patches were beneficial and expressed a willingness to continue using them which may have contributed to adherence.	*I didn't realise how lucky I was until the pain was under some control, which took almost a week, and then I realised how much the lidocaine was doing for me." (Patient 1, interview)*<br><br>*"I felt I'd won the lottery." (Patient 2, interview)*<br><br>*"I was in hospital for a week, and I came out with about four or five patches, and I was quite sad when I didn't have any more, because the pain went on." (Patient 6, interview)*
Patches and other procedures/medications	*"We were doing the randomisation, getting everything ready, consented and everything, I didn't even have… there was the anaesthetist who was already preparing for an SA block, so he didn't even give me time to put on the actual patches, because he eventually went immediately onto SA block." (Clinician, focus group 2)*<br><br>*"The only thing that I can think of is we had a patient in resus who had… put two or three patches on, then had a chest drain, and then was transferred up to [city]. So the positioning of the patches I am not sure what ended up happening to that… to the patches, because of the chest drain, So I don't know if the patches needed to be removed." (Clinician, focus group 2)*

Mixed views on the benefits of lidocaine patches (equipoise), lack of pain management options for older adults, and perceived safety of patches.	*"I think it comes from a sense of we're running out of options for these particular patients, let's go with lidocaine because we know it works for other types of pain of that age group." (Clinician, focus group 1)*<br><br>*"So, there's definitely not a feeling people are convinced that it's amazing… I don't think there's an overwhelming sense that it does great work, but I think it's something else we can offer people when we know that we can't offer them what we would our younger cohort," (Clinician, interview 1)*<br><br>*"I think we were disappointed whenever they were randomised to not getting one [patch]." (Clinician, focus group 2)*<br><br>*"I like the lidocaine patches, not going to lie, so I'd like everybody to get them." (Clinician, focus group 2)*<br><br>*"I know that there is… they [trauma consultants] push for lidocaine patches because they believe in it." (Clinician, interview 3)*<br><br>*"I think clinicians and patients are not in equipoise about this anymore, because when they get the patch they love it, because it's cold, they put it on and it's cold, it's over the area where the pain is, it's as if someone is doing something automatically. Whether it works or not I don't know, I suspect it does work." (Clinician, focus group 1)*<br><br>*"I think clinicians are very bought into giving the patches, as certainly we are here, that we do, and we automatically do that, and we advocate for it." (Clinician, focus group 2)*
Increase in awareness and belief in the benefits of lidocaine patches.<br>- But recognition need for evaluative research.	*"I think we just need to be mindful… it's an area of increasing focus, and that may change people's behaviours, it may change the way hospitals are reacting to patients with chest wall trauma… looking forward to the next trial people are already adapting the way they manage such patients on the basis of either no evidence or beginnings of evidence but no the definitive trial." (Clinician, focus group 2)*
Potential role of a placebo patch to overcome equipoise issues.	*"Whether it's the placebo effect of the actual application that is seeing the patient experience a benefit from whether it's actually the application of lidocaine topically, and I don't know the answer to that. I've had anecdotally people say, "That feels wonderful," well it's literally been on three seconds, so it couldn't possibly be doing any benefit yet." (Clinician, focus group 1)*<br><br>*"The doctor would want the lidocaine patch - randomised for it or not, he would still want it because that's our policy… maybe*

	that's where your placebo comes in, whether you could do randomisation to either you don't… you get it or you get placebo." (Clinician, focus group 2)

Clout M, *et al. Emerg Med J* 2024; 41:522–531. doi: 10.1136/emermed-2024-213905
